# Neuromusculoskeletal model that walks and runs across a speed range with a few motor control parameter changes based on the muscle synergy hypothesis

**DOI:** 10.1038/s41598-018-37460-3

**Published:** 2019-01-23

**Authors:** Shinya Aoi, Tomohiro Ohashi, Ryoko Bamba, Soichiro Fujiki, Daiki Tamura, Tetsuro Funato, Kei Senda, Yury Ivanenko, Kazuo Tsuchiya

**Affiliations:** 1 Department of Aeronautics and Astronautics, Graduate School of Engineering, Kyoto University, Kyoto daigaku-Katsura, Nishikyo-ku, Kyoto 615-8540 Japan; 2 Department of Life Sciences, Graduate School of Arts and Sciences, The University of Tokyo, 3-8-1 Komaba, Meguro-ku, Tokyo 153-8902 Japan; 3 Department of Mechanical Engineering and Intelligent Systems, Graduate School of Informatics and Engineering, The University of Electro-Communications, 1-5-1 Choufugaoka, Choufu-shi, Tokyo 182-8585 Japan; 4 Laboratory of Neuromotor Physiology, IRCCS Santa Lucia Foundation, 00179 Rome, Italy

## Abstract

Humans walk and run, as well as change their gait speed, through the control of their complicated and redundant musculoskeletal system. These gaits exhibit different locomotor behaviors, such as a double-stance phase in walking and flight phase in running. The complex and redundant nature of the musculoskeletal system and the wide variation in locomotion characteristics lead us to imagine that the motor control strategies for these gaits, which remain unclear, are extremely complex and differ from one another. It has been previously proposed that muscle activations may be generated by linearly combining a small set of basic pulses produced by central pattern generators (muscle synergy hypothesis). This control scheme is simple and thought to be shared between walking and running at different speeds. Demonstrating that this control scheme can generate walking and running and change the speed is critical, as bipedal locomotion is dynamically challenging. Here, we provide such a demonstration by using a motor control model with 69 parameters developed based on the muscle synergy hypothesis. Specifically, we show that it produces both walking and running of a human musculoskeletal model by changing only seven key motor control parameters. Furthermore, we show that the model can walk and run at different speeds by changing only the same seven parameters based on the desired speed. These findings will improve our understanding of human motor control in locomotion and provide guiding principles for the control design of wearable exoskeletons and prostheses.

## Introduction

Humans walk and run in accordance with the desired speed and circumstances. These gaits have different characteristics at the kinematic level. For example, the most notable differences are in the existence of a double-stance phase in walking, in which both feet are in contact with the ground, and a flight phase in running, in which both feet are in the air. Also, the center of mass (COM) moves differently at different gaits–at the mid-stance phase, it reaches its highest position during walking and its lowest position during running^1^. Many kinematic parameters, such as stride length and gait cycle, also change at different gaits and speeds^2^. At the kinetics level, the vertical ground reaction force shows a two-peaked shape for walking and a single-peaked shape for running^3–5^. Such differences are motor outcomes of the complicated musculoskeletal system controlled by the central nervous system (CNS).

Locomotor behavior is generated by propelling the body over the ground using the legs. However, the body has more degrees of freedom (DOF) in the joints than is necessary for this propulsion. Furthermore, the muscles have more DOF than the joints due to antagonistic pairs of muscles and multiarticular muscles. This means that humans use numerous and redundant DOF for different gaits and speeds. Although it is obvious that such redundancy plays an important role for adaptive locomotor behavior, it remains unclear how the CNS manipulates such a large number of DOF. The complex and redundant nature of the musculoskeletal system and various differences in the motor outcomes lead us to imagine that the motor control in the CNS is extremely complicated and that different gaits require different control strategies.

**Figure 1:**
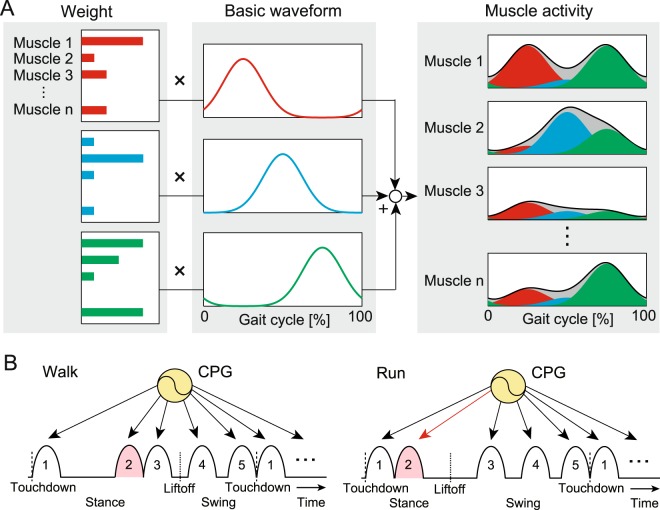
Schematic model of muscle synergy: (**A**) Muscle activities are explained by the linear combination of a small number of basic waveforms. In most cases, human walking and running can be explained by five waveforms, but only three waveforms are shown here to simplify the illustration. (**B**) Hypothetical CPG motor control model producing five activation pulses for motor commands. Depending on the gait, the model shifts the activation timing of the second pulse.

A large number of muscles contribute to generation of human movement, and they show complex activation patterns. However, an analysis of electromyographic (EMG) data shows that the linear combination of a small number of basic waveforms reproduces a large portion of the EMG patterns (Fig. 1A). This suggests that motor control in the CNS utilizes this low-dimensional structure to solve the motor redundancy problem (i.e., muscle synergy hypothesis)^6–12^ and that central pattern generators (CPGs) in the spinal cord are responsible for controlling this low-dimensional structure. Although the muscle synergy hypothesis remains an open question^13^, it provided fruitful insights for motor control mechanisms. In particular, the linear combination of five basic waveforms reproduces a large portion of the EMG patterns in both walking and running^14,15^, and the major difference between the gaits appears in the phase shift of the second basic waveform^14^. This phase shift also appears to change the running speed^14^. These findings suggest that five activation pulses produce a large portion of motor commands in human walking and running and that the generation timing of the second pulse plays an important role in determining the gait and speed (Fig. 1B). This physiological hypothesis implies that the major motor control structure is actually simple and shared and that the difference between the gaits and speeds can be explained by a few components.

Although physiological studies provide meaningful insights for the underlying neural mechanisms for motor skills in humans, it is difficult to fully clarify them using only analysis of experimental data. To overcome such limitations imposed by a single perspective, modeling studies have recently attracted attention because physiological findings enable us to construct reasonably realistic motor control models and investigate their functional roles through the model structure and parameters. Furthermore, because locomotor behavior is well-organized behavior generated through dynamic interactions between the CNS motor control system, musculoskeletal system, and environment, investigating motor control models by integrating them with sophisticated musculoskeletal models has allowed us to approach deeper neural mechanisms^16–25^. In particular, Taga^25^ and Hase *et al*.^20^ developed motor control models using neural oscillators^26^. Taga’s model had over 100 parameters and produced walking in a two-dimensional musculoskeletal model over a speed range from 0.7 to 1.2 m/s by changing only the constant neural drive to the oscillators. Hase *et al*.’s model had 125 parameters and produced both walking and running in a three-dimensional musculoskeletal model by changing the parameters through evolutionary computation. In contrast, Song and Geyer^23,24,27^ developed a reflex-based motor control model. More specifically, their model in^23^ had 82 parameters and produced walking and running in a three-dimensional musculoskeletal model by changing the parameters through an optimization technique. In^24^, they constrained their musculoskeletal model to the sagittal plane and showed that the running speed can be changed from 2.4 to 4.0 m/s by linearly changing only the motor control parameters based on the desired speed (in this case, they used 64 parameters). Furthermore, they integrated a motor control model with 30 parameters into a two-dimensional musculoskeletal model in^27^ and demonstrated that the walking speed can be changed from 0.8 to 1.8 m/s by changing only nine key parameters among the 30 parameters. Günther and Ruder^19^ developed a motor control model based on the *λ* model of the equilibrium-point hypothesis^28^. Their model had about 20 parameters and changed the walking speed from 0.8 to 1.1 m/s in a two-dimensional musculoskeletal model by changing the stretch reflex feedback gain and trunk reference angle.

In contrast with these motor control models, Neptune *et al*.^16,22^, Jo and Massaquoi^21^, and our previous work^17^ developed motor control models for walking based on the muscle synergy hypothesis. In particular, Neptune *et al*.’s model used five activation pulses, whose shapes were determined from the analysis of measured EMG data. They found the walking solution of a complete gait cycle by optimizing the onset, duration, and magnitude of the pulses in two-dimensional^22^ and three-dimensional^16^ musculoskeletal models. Jo and Massaquoi’s model had a linear combination of four activation pulses and the regulation of the COM position and trunk pitch angle. They changed the walking speed from 0.6 to 1.4 m/s in a two-dimensional musculoskeletal model by changing the gait cycle duration, magnitude of the pulses, and COM reference. Our previous model^17^ had 69 parameters and involved the linear combination of five activation pulses to produce walking in a two-dimensional musculoskeletal model. However, muscle synergy-based motor control models have not demonstrated different gaits and speeds. The present study aims to demonstrate that it is possible to generate walking and running with different speeds in the context of the muscle synergy hypothesis from a dynamic viewpoint through forward dynamic simulation using our previous neuromusculoskeletal model. More specifically, we generated different gaits using only seven key motor control parameters selected from the physiological findings and hypothesis. Furthermore, we showed that the gait speed can be changed by changing the same seven parameters based on the desired speed. Finally, we evaluated important roles of the muscle synergy-based low-dimensional structure in the motor control of human locomotion.

## Results

### Model

**Figure 2:**
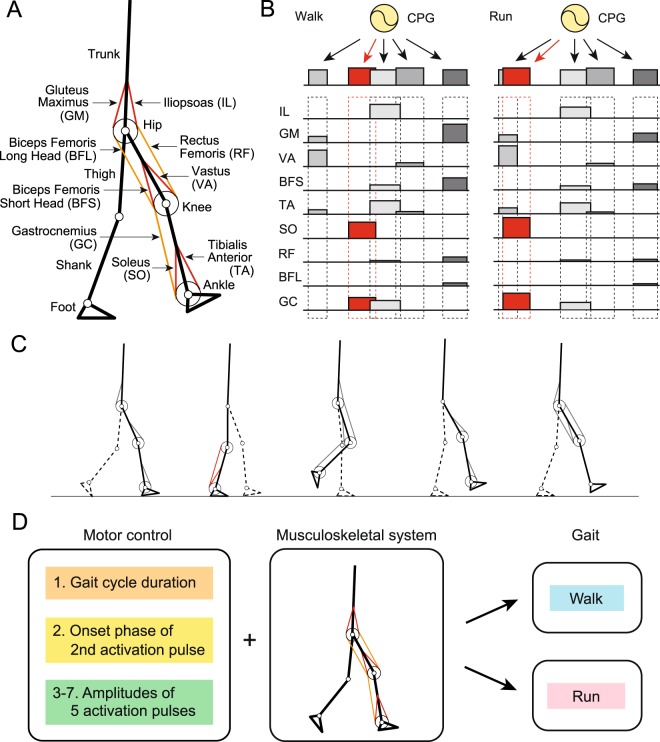
Neuromusculoskeletal model for human walking and running: (**A**) musculoskeletal model, (**B**) motor command in the movement generator composed of the linear combination of five rectangular pulses based on hypothetical motor program, (**C**) muscles activated by each of the five rectangular pulses, and (**D**) seven motor control parameters to produce walking and running through the musculoskeletal model.

Our musculoskeletal model has seven rigid links and nine principal muscles for each leg (Fig. 2A), which are driven by command signals from the motor control model. This model is two-dimensional, and only the sagittal plane motion is modeled. The motor control model consists of two components: one is the movement generator, which produces five weighted activation pulses (rectangular pulses) in a feedforward fashion based on the muscle synergy hypothesis (Fig. 2B,C), and the other is the movement regulator, which regulates the locomotion movement in a feedback fashion based on somatosensory information with transmission delay. This control model has 69 parameters (61 for the movement generator and eight for the movement regulator). We first determined these parameters for walking at one desired speed based on our previous work^17^, which succeeded in the generation of walking. We then used different values for only seven parameters to change from walking to running for the desired speed based on the physiological findings and hypothesis^14^ (Fig. 2D). One of these key parameters is the gait cycle duration, which determines the time interval between successive sets of the five activation pulses. Another is the phase of the second activation pulse within one gait cycle, which stems from the hypothetical motor program (Fig. 2B) obtained from the major difference between the gaits by the muscle synergy analysis^14^. The other five key parameters are the amplitudes of the five activation pulses for delivery to the muscles. To change the gait speed, we varied the same seven parameters depending on the desired speed based on the results of the muscle synergy analysis^14^.

### Simulations of walking and running

**Figure 3:**
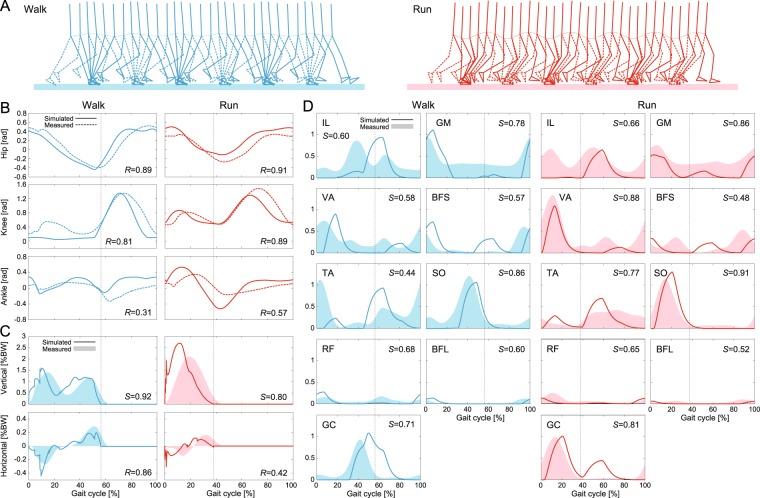
Simulated locomotor behavior for 1.6 m/s of desired speed for walking and running: (**A**) simulation stick diagrams (also see Movies S1 and S2) and comparison of simulated and measured data for (**B**) joint movements, (**C**) ground reaction forces, and (**D**) muscle activities. Vertical dotted lines indicate the liftoff timing in the simulated locomotion. Increasing joint angle corresponds to joint flexion. Measured data were obtained at a belt speed of 1.85 m/s. *R* is correlation coefficient and *S* is cosine similarity.

**Figure 4:**
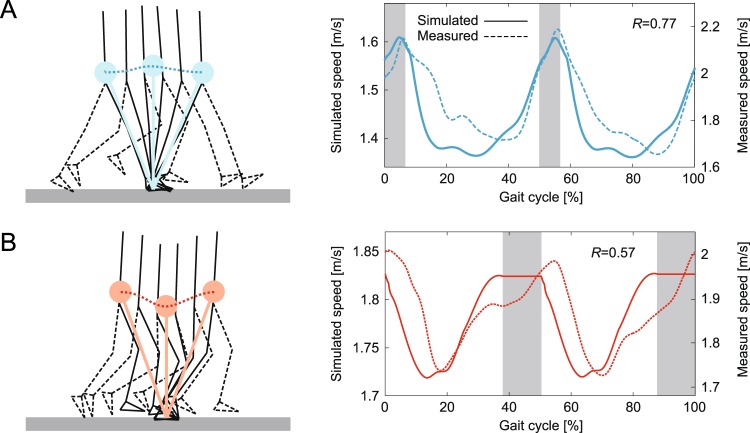
Simulated COM movement and horizontal speed for 1.6 m/s of desired speed for (**A**) walking and (**B**) running. Estimated horizontal COM speeds from the measured data at a belt speed of 1.85 m/s are also shown in the right panels. Gray regions indicate simulated double-stance phase for walking and flight phase for running. *R* is correlation coefficient and *S* is cosine similarity.

**Figure 5:**
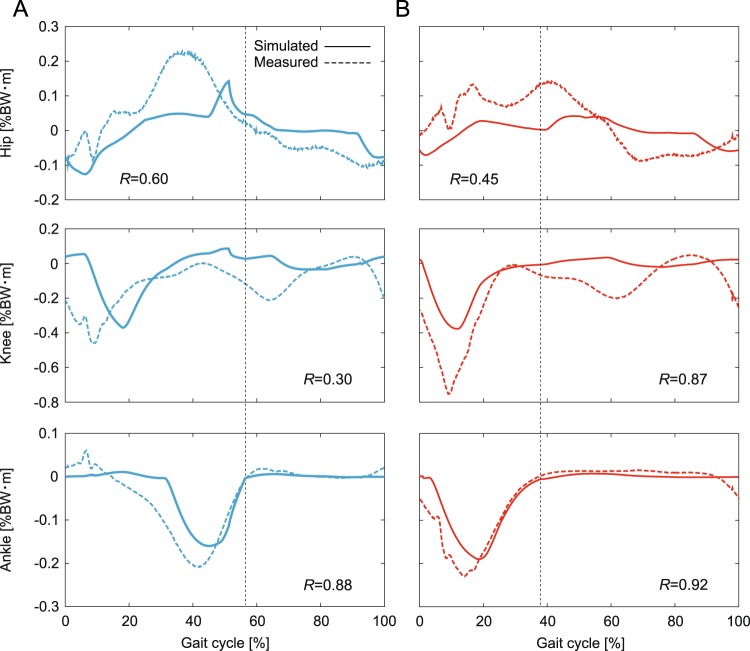
Simulated joint torques by muscles for 1.6 m/s of desired speed and estimated joint torques from the measured data at a belt speed of 1.85 m/s for (**A**) walking and (**B**) running. Vertical dotted lines indicate the liftoff timing in the simulated locomotion. Increasing joint angle corresponds to joint flexion. *R* is correlation coefficient.

The integrated neuromusculoskeletal model produced walking and running behavior for the desired speed of 1.6 m/s, as shown in Fig. 3A. The generated average speed was 1.45 m/s for walking and 1.78 m/s for running (Fig. 4). The simulated walking has double-stance phases, while the simulated running has flight phases. Animations of the gaits are available in Movies S1 and S2. The simulation results were compared with data measured during human walking and running. Figure 3B–D show the comparison of joint angles, ground reaction forces, and muscle activities, respectively, for one gait cycle, where *S* is the cosine similarity used for positive value factors and *R* is the correlation coefficient used for the other factors. Figures 4 and 5 show the comparison of the simulated COM movements and joint torques, respectively, with those estimated from measured data. The simulation results capture the kinematic and dynamic characteristics of human gaits. In particular, the hip and knee joints had high agreement (*R* = 0.89 and 0.89 for walking, *R* = 0.91 and 0.81 for running). The vertical ground reaction force showed a two-peaked shape for walking (*S* = 0.92), while it showed a one-peaked shape for running (*S* = 0.80), which matched the characteristic difference observed in human gaits^3–5^. In addition, the simulated walking had larger forces in the horizontal direction (*R* = 0.86), and the simulated running had larger forces in the vertical direction, as observed in humans. Furthermore, the phase shift of the second activation pulse established activation timings of soleus (SO) and gastrocnemius (GC) muscles similar to those of the measured data for walking and running (*S* = 0.86 and 0.91 for SO, *S* = 0.71 and 0.81 for GC), which produced high agreement in the ankle joint torque (*R* = 0.88 for walking, *R* = 0.92 for running).

However, our model was limited in its ability to accurately reproduce the locomotor behavior observed in humans. At the kinematics level, the stance leg was almost straight with a small knee flexion in the early stance phase of walking. Vastus (VA) muscles are suggested to play an important role for the early-stance knee flexion in walking^29^. In our model, larger activation of VA muscles (*S* = 0.58) prevented knee flexion and caused a discrepancy in the knee joint torque (*R* = 0.30). The straight-leg walking caused larger peaks of ground reaction forces, as observed in the previous modeling study using a musculoskeletal model^29^. In addition, our model showed larger dorsiflexion of the ankle joint in the swing phase of walking (*R* = 0.31) due to larger activation of tibialis anterior (TA) muscles (*S* = 0.44) and showed larger plantarflex in the early swing phase of running (*R* = 0.57) due to larger activation of GC muscles. Larger activation of GC muscles was also observed in the early swing phase of walking. Moreover, larger activation of biceps femoris short head (BFS) muscles appeared in the early swing phase of both walking and running (*S* = 0.57 for walking, *S* = 0.48 for running). Iliopsoas (IL) muscle activations had low agreement (*R* = 0.60 for walking, *R* = 0.66 for running) partly because of the difficulty of accurate measurement because it is a deep muscle^30^, which caused a discrepancy in the hip joint torque (*R* = 0.60 for walking, *R* = 0.45 for running). Furthermore, although the generated gait speed had patterns similar to those in measured data (*R* = 0.77 for walking, *R* = 0.57 for running), our model could not achieve the desired speed (1.6 m/s). In other words, walking was slower (1.45 m/s on average) and running was faster (1.78 m/s on average) than desired. Although we used the TA and SO muscles as a speed regulator, their contribution was limited to only a few percent for the formation of muscle activation patterns (3.9% for walking, 3.7% for running). Although even a small contribution can play an important role for speed regulation^17^, it was not sufficient to achieve the desired speed. Moreover, the liftoff timings were slightly earlier, and the ground reaction forces fluctuated because four discrete points on each sole were used for the foot contact model.

### Simulations for different speeds

**Figure 6:**
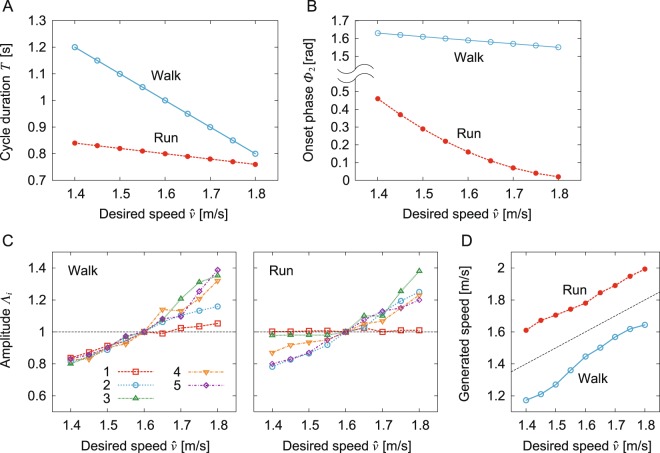
Simulated change of gait speed: (**A**) gait cycle duration, (**B**) onset phase of second activation pulse for desired speed, (**C**) amplitudes of five activation pulses relative to those for a desired speed of 1.6 m/s, and (**D**) generated speed.

Figure 6 shows the simulation results of changing the speed for each gait. We searched the amplitudes of the five activation pulses so that the model achieved periodic gaits under the condition that the gait cycle duration (Fig. 6A) and onset phase of the second activation pulse (Fig. 6B) varied for the desired speed in a fashion similar to those of humans^14^ (the gait cycle duration undergoes a larger change while walking and the phase of the second basic waveform exhibits a larger change while running). As a result, the amplitudes of the pulses generally increased for the desired speed (Fig. 6C), which increased the gait speed in a way similar to that of humans (Fig. 6D).

Although our model achieved different speeds for each gait, the speed range was limited (1.2–1.6 m/s for walking, 1.6–2.0 m/s for running). The walking speeds were moderate to fast and the running speeds were relatively slow and close to walk–run transition speeds in humans^31^. Our model did not achieve slower walking or faster running. In addition to the small contribution of the TA and SO muscles to speed regulation, a lack of diversity in motor command patterns due to the linear combination of simple rectangular pulses may prevent our model from achieving these gaits. Moreover, the motor commands by the linear combination worked in a feedforward manner. Because sensory-motor coordination is crucial for adapting to various situations and changing speeds, incorporating reflex models is important^18,23,25,32,33^. The timing manipulation of the activation pulses based on kinematic events and sensory information would be useful^8,34^. In addition, although it is generally accepted that similar muscle synergy structures are used for different speeds in each gait^8,14,35,36^, it was reported that fast and slow walking and also fast and slow running have some structural differences, such as the number of basic waveforms^37^. Furthermore, instability is another possible factor that prevents slower walking, as many physical models have suggested that it is difficult to maintain dynamic stability at slow speeds in walking^38–40^. The restriction of our musculoskeletal model to the sagittal plane and the simplified upper body are also possible factors that prevent faster running. For example, the pelvis motion in the transverse plane contributes to maximizing horizontal propulsion^41^ and swinging the arms contributes to generating vertical propulsion in running^42^. We will introduce such additional factors to improve our neuromusculoskeletal model in the future.

## Discussion

Previous studies have proposed various motor control models with a large number of parameters based on physiological hypotheses. They showed the ability to produce walking and running and change gait speed by changing a large number of parameters through forward dynamic simulations of the human musculoskeletal system^20,23,24^ (some studies showed that a few among many motor control parameters can change the walking speed^19,25,27^). Although our motor control model also had a large number of parameters (=69), changing only seven key parameters among the 69 enabled our musculoskeletal model to walk and run. Furthermore, the musculoskeletal model changed the speed of each gait by changing only the same seven parameters based on the desired speed. Although other parameter choices could also generate different gaits and speeds, our results were due to the simple and shared control scheme for different gaits and speeds in our motor control model based on the muscle synergy hypothesis. Motor commands in our model were mainly generated by the linear combination of five activation pulses, and the main differences between the gaits and speeds were the onset phase of the second activation pulse and the amplitudes of the activation pulses. The use of such a low-dimensional motor control structure makes the gait generation and selection simple. In particular, humans change their gait almost within one gait cycle^14^, which requires a rapid change in the motor planning. The simple and common structure between the gaits substantially lessens the computational burden for generating motor commands, and the motor control needs to only specify when and how much the activation pulses are recruited. However, even if the gait transition strategy at the motor control level is simple, this task remains difficult because the musculoskeletal dynamics must be consistent with the gait transition context (e.g., different gaits have different basins of attraction). Furthermore, reduced gravity induces smooth gait transitions without abrupt changes in either intensity or timing of EMG patterns^43^, and the underlying mechanism in these gait transitions remains unclear. We intend to investigate them from a dynamic viewpoint using our neuromusculoskeletal model in the future.

The temporal profile of each joint angle does not necessarily have a large difference between the gaits, as shown in Fig. 3B. Instead, some specific locomotion factors explained by the combination of the joint angles show crucial differences, and these factors distinguish different gaits. One factor is the existence of double-stance and flight phases for walking and running, respectively, as shown in Fig. 3A. Another is the COM movement, as shown in Fig. 4. In human walking, although there is knee flexion in the early stance phase, the stance leg is almost straight and it rotates around the foot contact point like an inverted pendulum^44^. Therefore, the COM is at its highest position during the mid-stance phase and at its lowest position during the double-stance phase. In contrast, the horizontal speed is lowest during the mid-stance phase and highest during the double-stance phase (these are confirmed from the simulation results in Fig. 4A). This means that the COM height changes in antiphase with the horizontal speed and that humans produce efficient walking through the pendular exchange of potential and kinetic energies while conserving mechanical energy^45–48^. In contrast, both the COM height and horizontal speed have their lowest value during the mid-stance phase and their highest value during the flight phase in human running (this is also confirmed from the simulation results in Fig. 4B). This means that the potential and kinetic energies move in phase, and running does not have an energy exchange, unlike walking. Instead, during the stance phase, these energies are stored in elastic elements of the body and released in the next step^49,50^. As a result, the COM moves like a mass-spring system. Thus, the COM dynamics clearly differs between the gaits. It has recently become widely accepted that both walking and running are explained by the compliant leg and that humans use different leg stiffnesses for different gaits and speeds^4,51,52^. Although the present study showed that a few parameters in the motor control system can produce these crucial differences in the COM movement, they have never been produced by the motor control model alone, but were produced for the first time through the dynamics of our musculoskeletal model, as in^20,23^. Although many previous modeling studies focused only on the motor control system and provided useful biological insights^53–55^, the integration of the musculoskeletal system is also important.

To focus on the different COM movements between the gaits, a model consisting of a mass point and two massless legs was used to investigate dynamic mechanisms in human locomotion by incorporating leg compliance by Geyer *et al*.^4^ and energetic optimization by Srinivasan and Ruina^56^ for motor control. Although it had only two DOF in the body dynamics (vertical and horizontal movements of the mass point), the model succeeded in the generation of walking and running behaviors with double-stance phases for walking (though infinitesimally in^56^) and flight phases for running and with different COM movements between the gaits through a few different parameters in their models, similar to our results. This was partly because of the model simplicity. In contrast, we used more biologically detailed musculoskeletal and neural control models, which introduced articulated skeletal structure, actuation by uniarticular and biarticular muscles, and motor-neuron activities. These models are able to produce biologically plausible behavior, such as multi-joint movement, foot action (heel contact, toe-off, and push-off), and balancing of the trunk. However, as the number of model elements, such as joints, muscles, and neural elements, increase to make the models more biologically plausible, the number of parameters increases significantly. This makes modeling studies difficult. The muscle synergy hypothesis enables a large reduction in the number of model parameters due to the assumption of a low-dimensional structure at the motor-neuron activation level, which will accelerate modeling studies and increase their importance for understanding motor control strategies. Furthermore, this physiological hypothesis is not limited to human walking and running. For example, in voluntary movements during human walking, such as kicking a ball and stepping over an obstacle, muscle activities are explained by the superposition of the basic waveforms for normal walking and an additional waveform timed to the voluntary task^57^. In unstable and unsteady conditions, basic waveforms widened to improve the robustness^58,59^. In the development from neonate to adult, the number of basic waveforms increases in parallel with the biomechanical development and the waveform shapes also change to achieve effective locomotor behavior^60^. Moreover, similar low-dimensional structures appear in animals, such as rats, cats, dogs, monkeys, and guineafowls, despite substantial phylogenetic distances and morphological differences^60–62^. Low-dimensional structures extracted from muscle synergy analysis have also provided meaningful insights for gait deficits due to neurological impairment. Stroke sufferers often have reduced motor performance through the merging of some basic waveforms, and furthermore the merged waveforms are split into multiple waveforms in the post-stroke recovery to improve the motor performance^36,63^. Persons with Parkinson’s disease require a lower-dimensional structure than that of healthy older persons, where the basic waveforms are altered while the weight distributions within waveforms are unaffected^64^. Although modeling studies have been performed through the integration of motor control and musculoskeletal models by focusing on motor tasks, animal species, development stages, and neurological impairments^65–72^, the muscle synergy hypothesis would help clarify dynamic reasons for common and distinct characteristics in these motor controls^73–76^.

Although muscle synergy analyses have provided fruitful insights for motor control mechanisms, as described above, the muscle synergy hypothesis remains an open question^13^. For example, muscle synergy structures were altered with an ankle exoskeleton and it is suggested that humans do not exclusively use low-dimensional control structures, especially when learning a new task, such as adapting to external assistance^77^. Furthermore, it was reported that low-dimensional structures extracted from muscle activities can be provided by biomechanical and task constraints rather than by neural activity^78,79^. However, neural bases for low-dimensional controls have been reported^9,80–85^. Our motor control model is simple and thus extensible for further investigation of motor control mechanisms. Understanding motor control mechanisms through modeling improves our understanding of the underlying dynamic mechanisms in locomotion, but it also provides a guiding principle for rehabilitation techniques, such as evaluation of rehabilitation strategies and development of rehabilitation tools and methods, and for the design of controls for wearable exoskeletons, prostheses, and legged robots^86–89^.

## Methods

### Modeling

We used the same two-dimensional musculoskeletal model (Fig. 2A) as that used in our previous work^17^, which was developed based on a previous musculoskeletal model^90^ whose physical parameters were determined from measured data of humans^91,92^. The skeletal part of our model has seven rigid links–trunk (head, arms, and torso) and thigh, shank, and foot of each leg–and nine DOF–hip, knee, and ankle joint angles of each leg and horizontal and vertical translations and rotation of the trunk. The joint angles were defined so that increasing joint angle corresponds to joint flexion. Each joint has a linear viscous element whose coefficient is determined based on^91^, and the knee and ankle joints are subject to large linear elastic and damping torques when these joint angles exceed their limits (0.1 to 2.8 rad for the knee and −1.0 to 0.54 rad for the ankle). The foot contact is modeled using four viscoelastic elements at each sole. The model uses nine principal muscles to achieve the necessary motions in each leg. Six muscles produce uniarticular motion: hip flexion (IL), hip extension (gluteus maximus [GM]), knee extension (VA), knee flexion (BFS), ankle flexion (TA), and ankle extension (SO). Three muscles produce biarticular motion: hip flexion and knee extension (rectus femoris [RF]), hip extension and knee flexion (biceps femoris long head [BFL]), and knee flexion and ankle extension (GC). The moment arms of the muscles around the joints are constant regardless of joint angle. The muscle model consists of contractile and passive elements. The contractile part depends on force-length and force-velocity relationships and the activation determined through a low-pass filter of motor commands from the neural motor control model. The equations of motion of this model were derived using Lagrangian equations and solved using the fourth-order Runge-Kutta method with time steps of 0.02 ms for the forward dynamic simulation.

The motor control is modeled based on the hypothetical motor program at the spinal cord level (movement generator) and the regulation of locomotion movement at the brainstem and cerebellum levels (movement regulator). This model is the same as that in our previous work^17^, in which we compared two models with and without sensory regulation by phase resetting, but the present model does not use sensory regulation.

The muscle synergy analysis showed that the linear combination of five basic waveforms explains a large portion of the muscle activation patterns in both human walking and running and the major difference is in the phase of the second waveform^14^. These findings suggest that five basic signals produce a large portion of motor commands in human walking and running and that the generation timing of the second signal plays an important role in determining the gait. Therefore, the movement generator uses five activation pulses for each leg (Fig. 2B), similar to the previous work^17,21,22^, and it changes the activation phase of the second pulse. More specifically, the phase of the activation pulses of our model is denoted using *ϕ* (0 ≤ *ϕ* < 2*π*), where $\dot{\varphi }=2\pi /T$ for gait cycle duration *T*. Because the basic waveforms are each characterized by a relatively narrow (Gaussian-like) peak of activation at a particular phase of the gait cycle in humans^8^ and the muscle activation is generated through a low-pass filter in our model, rectangular pulses *p*_*i*_ (*ϕ*) (*i* = 1, …, 5) are used for activation:

1 $${p}_{i}(\varphi )=\{\begin{array}{ll}1 & {{\rm{\Phi }}}_{i} < \varphi \le {{\rm{\Phi }}}_{i}+{{\rm{\Delta }}}_{i}\\ 0 & {\rm{otherwise}}\end{array}\,i=1,\ldots ,5$$

where Φ_*i*_ and Δ_*i*_ (*i* = 1, …, 5) are the onset phase and duration, respectively, of the rectangular pulses. We used different values for the gait cycle duration *T* and phase of the second activation pulse Φ_2_ to change from walking to running (Fig. 6A,B). Although the amplitudes of muscle activation patterns differ depending on the gait and speed, the muscle synergy analysis shows that the ratio between muscles in the weighting coefficients of the basic waveforms is consistent across different speeds for both walking and running, as in^14^. Based on this, we determined the muscle synergy-based motor command ${u}_{m}^{{\rm{Syn}}}$ (*m* = IL, GM, VA, BFS, TA, SO, RF, BFL, and GC) by

2 $${u}_{m}^{{\rm{Syn}}}=\sum _{i=1}^{5}\,{{\rm{\Lambda }}}_{i}{w}_{m,i}{p}_{i}(\varphi )$$

where *w*_*m*,*i*_ (*i* = 1, …, 5) is the weighting coefficient of five activation pulses to motor neurons (*w*_*m*,*i*_ ≥ 0) and Λ_*i*_ (*i* = 1, …, 5) is the tuning parameter of the amplitude for different gaits and speeds (Fig. 6C). The motor commands by these activation pulses are identical between the legs except for the antiphase behavior (*ϕ* for one leg and *ϕ* + *π* for the other leg). The movement generator has 61 parameters in total (*T*, Φ_*i*_, Δ_*i*_, *w*_*m*,*i*_, and Λ_*i*_). Only seven parameters (*T*, Φ_2_, and Λ_1, …, 5_) were given different values for the generation of different gaits and speeds.

In addition to the muscle synergy-based control, we used the movement regulator to regulate the locomotion movement based on somatosensory information, where only two crucial factors are incorporated for simplicity: maintenance of an upright posture and desired forward locomotion speed. For the maintenance of an upright posture, a simple feedback control regulates the balance of the trunk pitch to prevent it from falling over using antagonistic uniarticular muscles in the hip of the standing leg.

3 $${p}_{m}^{{\rm{Trunk}}}=\{\begin{array}{ll}-{\kappa }_{m}(\theta -\hat{\theta })-{\sigma }_{m}\dot{\theta } & {\rm{in}}\,{\rm{stance}}\,{\rm{phase}}\\ 0 & {\rm{otherwise}}\end{array}$$

where *θ* is the trunk pitch angle, $\dot{\theta }$ is the trunk pitch angular rate, $\hat{\theta }$ is the reference angle, and *κ*_*m*_ and *σ*_*m*_ are the gain parameters (*κ*_*m*_ = *σ*_*m*_ = 0 when *m* ≠ IL or GM). For the maintenance of the locomotion speed, a simple feedback control is used to increase the ankle push-off when the speed is lower than desired and suppress the pushing force in the opposite case by antagonistic uniarticular muscles in the ankle of the standing leg.

4 $${p}_{m}^{{\rm{Speed}}}=\{\begin{array}{ll}-{\lambda }_{m}(v-\hat{v}) & {\rm{in}}\,{\rm{stance}}\,{\rm{phase}}\\ 0 & {\rm{otherwise}}\end{array}$$

where *v* is the forward locomotion speed, $\hat{v}$ is its desired value, and *λ*_*m*_ is the gain parameter (*λ*_*m*_ = 0 when *m* ≠ TA or SO). Because the movement regulator is at the brainstem and cerebellar levels, the command signals are delayed and the motor command ${u}_{m}^{{\rm{R}}{\rm{e}}{\rm{g}}}$ is given by

5 $${u}_{m}^{{\rm{Reg}}}(t)={p}_{m}^{{\rm{Trunk}}}(t-\tau )+{p}_{m}^{{\rm{Speed}}}(t-\tau )$$

where *τ* (=80 ms) is the delay in receiving transmission of somatosensory information at the brainstem and cerebellar levels and sending the motor command to the spinal cord level. The movement regulator has eight parameters in total: *κ*_IL_, *κ*_GM_, $\hat{\theta }$, *σ*_IL_, *σ*_GM_, *λ*_TA_, *λ*_SO_, and $\hat{v}$. $\hat{v}$ is the desired speed and the other control parameters remain unchanged regardless of the gait and speed.

The motor output is given by the summation of these two components from the movement generator and movement regulator.

6 $${u}_{m}={u}_{m}^{{\rm{Syn}}}+{u}_{m}^{{\rm{Reg}}}$$

### Model parameters

To produce walking and running, 69 parameters in the motor control model have to be determined. In this study, we first determined the parameters for walking at one desired speed based on our previous work^17^, which had already succeeded in the generation of walking at 1.3 m/s, and then modified the parameters to achieve running. Specifically, we used Λ_*i*_ = 1 (*i* = 1, …, 5) for walking with the desired speed $\hat{v}=1.6$ m/s and the gait cycle duration *T* = 1.0 s based on^14^ and determined the other parameters by hand-tuning the previous result as follows: the onset phase and duration of activation pulses Φ_1_ = 0 rad, Φ_2_ = 1.59 rad, Φ_3_ = 2.44 rad, Φ_4_ = 3.69 rad, Φ_5_ = 5.36 rad, Δ_1_ = 0.75 rad, Δ_2_ = 1.10 rad, Δ_3_ = 1.20 rad, Δ_4_ = 1.07 rad, and Δ_5_ = 0.94 rad; the weighting coefficients of the activation pulses *w*_GM,1_ = 0.33, *w*_VA,1_ = 1.02, *w*_TA,1_ = 0.27, *w*_SO,2_ = 1.09, *w*_GC,2_ = 0.84, *w*_IL,3_ = 0.99, *w*_BFS,3_ = 0.34, *w*_TA,3_ = 0.97, *w*_RF,3_ = 0.02, *w*_GC,3_ = 0.65, *w*_VA,4_ = 0.23, *w*_TA,4_ = 0.15, *w*_GM,5_ = 1.17, *w*_BFS,5_ = 0.76, *w*_RF,5_ = 0.29, *w*_BFL,5_ = 0.14, and the other *w*_*m*,*i*_ = 0; the gain parameters for the movement regulator *κ*_IL_ = −1.0, *κ*_GM_ = 2.0, *σ*_IL_ = −0.2, *σ*_GM_ = 0.4, *λ*_TA_ = −0.2, and *λ*_SO_ = 0.04; and the reference trunk pitch angle $\hat{\theta }\mathrm{=0.01}$ rad. After establishing the walking parameters, we changed seven parameters as follows to achieve running at the same desired speed: *T* = 0.8 s, Φ_2_ = 0.16 rad, Λ_1_ = 1.34, Λ_2_ = 1.29, Λ_3_ = 0.73, Λ_4_ = 0.62, and Λ_5_ = 0.50. The gait cycle duration was determined based on that in humans^14^ and the other six parameters (Φ_2_ and Λ_1, …, 5_) were determined by hand-tuning the result of walking so that the model achieved running. We decided that the model had achieved walking or running when the model kept walking or running without falling over for at least 10 steps.

We changed the speed of the model for each gait by changing the seven parameters from the values obtained for the desired speed $\hat{v}$ = 1.6 m/s. More specifically, we first changed the gait cycle duration *T* (Fig. 4A) and phase of the second activation pulse Φ_2_ (Fig. 4B) for the desired speed $\hat{v}$ in a fashion similar to those of humans^14^, where the gait cycle duration has a larger change in walking and the phase of the second basic waveform has a larger change in running. Under these conditions, we then determined five parameters (amplitudes of five activation pulses Λ_1, …, 5_) by hand-tuning the results for the desired speed $\hat{v}\mathrm{=1.6}$ m/s so that the model achieved walking or running for the different desired speeds $\hat{v}$ (Fig. 4C).

### Human data measurement

To compare the simulation results with the locomotor behavior observed in human gaits, kinematic, ground reaction force, and muscle EMG data were collected from one healthy man (age 22, weight 63.3 kg, and height 166 cm) as he walked or ran for 60 s on a split-belt treadmill (ITR3017, Bertec Corp.) equipped with two embedded force plates at a belt speed of 1.85 m/s (we used this speed because it is close to walk–run transition speeds in humans^31^). The experimental protocols were approved by the Ethics Committee of Doshisha University, and the participant gave written informed consent prior to data collection according to Doshisha University procedures.

Kinematic data were measured with a motion capture system (Mac 3D Digital RealTime System, Motion Analysis Corp.). The motion capture and force plate sampling rates were set at 500 Hz. Reflective markers were attached to the participant at the following locations: the head and bilaterally on the upper limit of the acromion, elbow, wrist, greater trochanter, lateral condyle of the knee, lateral malleolus, second metatarsal head, and heel. The hip, knee, and ankle joint angles and horizontal COM speed were calculated from the measured kinematic data. The measured force data were low-pass filtered using a second-order Butterworth filter with a cut-off of 10 Hz. EMG data were collected from 16 muscles on the right side of the subject’s body: IL, GM, VA, TA, SO, RF, GC, biceps femoris (BF), adductor longus (ADD), sartorius (SART), erector spinae at L2 (ESL2), peroneus longus (PERL), semitendinosus (ST), external oblique (OE), rectus abdominis superior portion (RAS), gastrocnemius medialis (GAM), and tensor fasciae latae (TFL). All data were collected using surface electrodes. Six EMG traces (SO, TA, VA, GC, BF, and RF) were recorded using Biolog DL-3100 (S&ME Corp.), five EMG traces (ADD, SART, IL, GM, and ESL2) were recorded using WEB-7000 (Nihon Kohden), and six EMG traces (PERL, ST, OE, RAS, GAM, and TFL) were recorded using FreeEMG (BTS Bioengineering). EMG data of the BF muscles were used for the comparison with the simulation results of BFS and BFL muscles. All EMG signals were recorded at a sampling rate of 1 kHz, high-pass filtered using a second-order Butterworth filter with a cut-off of 1 Hz, demeaned, rectified, and low-pass filtered using a zero-lag Butterworth filter with a cut-off of 5 Hz.

The gait cycle duration was defined as starting with touchdown of the right leg and ending with the next touchdown of the right leg, and these touchdown timings were determined by the value of vertical ground reaction force. After separating the motion, force, and EMG data by cycles, the data for one cycle were arranged to be 500 points, and their averages were calculated. These data were verified to be representative of measured data for human walking and running by comparison with those of eight healthy subjects in our previous work^14^. For comparison with the simulated muscle activations, we determined the magnitude of the measured EMG data so that the average of the maximum EMG values between walking and running was identical to that of the simulation results for each muscle. Under these conditions, we estimated joint torques based on our muscle model by using measured kinematic and EMG data. To evaluate the similarity between the simulation results and measured data, we used the cosine similarity *S* for the vertical ground reaction forces and muscle activities that have positive values, and used the correlation coefficient *R* for the other data.

## Supplementary information

Supplementary Information

Movie S1

Movie S2
