# Supplementary material for: Neuromusculoskeletal model that walks and runs across a speed range with a few motor control parameter changes based on the muscle synergy hypothesis
Source: Sci Rep. 2019 Jan 23;9:369. doi: 10.1038/s41598-018-37460-3 (PMC6344546; doi:10.1038/s41598-018-37460-3)
Supplement: Supplementary file 1 — Supplementary Information [file 41598_2018_37460_MOESM1_ESM.pdf]

Supplementary Information:
Neuromusculoskeletal model that walks and runs across a speed range with a few motor control parameter changes based on the muscle synergy hypothesis

Shinya Aoi[1,*], Tomohiro Ohashi[1], Ryoko Bamba[1], Soichiro Fujiki[2], Daiki Tamura[1], Tetsuro Funato[3], Kei Senda[1], Yury Ivanenko[4], and Kazuo Tsuchiya[1]

[1]Dept. of Aeronautics and Astronautics, Graduate School of Engineering, Kyoto University, Kyoto daigaku-Katsura, Nishikyo-ku, Kyoto 615-8540, Japan
[2]Dept. of Life Sciences, Graduate School of Arts and Sciences, The University of Tokyo, 3-8-1 Komaba, Meguro-ku, Tokyo 153-8902, Japan
[3]Dept. of Mechanical Engineering and Intelligent Systems, Graduate School of Informatics and Engineering, The University of Electro-Communications, 1-5-1 Choufugaoka, Choufu-shi, Tokyo 182-8585, Japan
[4]Laboratory of Neuromotor Physiology, IRCCS Santa Lucia Foundation, 00179, Rome, Italy
[*]shinya_aoi@kuaero.kyoto-u.ac.jp

Supplementary movies

We prepared two supplementary movies to show the simulated locomotor behavior:

S1. Simulated walking at a desired speed of 1.6 m/s.

S2. Simulated running at a desired speed of 1.6 m/s.
